# Meeting the Healthy People 2020 Objectives to Reduce Cancer Mortality

**DOI:** 10.5888/pcd12.140482

**Published:** 2015-07-02

**Authors:** Hannah K. Weir, Trevor D. Thompson, Ashwini Soman, Bjorn Møller, Steven Leadbetter, Mary C. White

**Affiliations:** Author Affiliations: Trevor D. Thompson, Steven Leadbetter, Mary C. White, Centers for Disease Control and Prevention, Atlanta, Georgia; Ashwini Soman, Northrop Grumman Corporation, Atlanta, Georgia; Bjorn Møller, Cancer Registry of Norway, Oslo, Norway.

## Abstract

**Introduction:**

Healthy People 2020 (HP2020) calls for a 10% to 15% reduction in death rates from 2007 to 2020 for selected cancers. Trends in death rates can be used to predict progress toward meeting HP2020 targets.

**Methods:**

We used mortality data from 1975 through 2009 and population estimates and projections to predict deaths for all cancers and the top 23 cancers among men and women by race. We apportioned changes in deaths from population risk and population growth and aging.

**Results:**

From 1975 to 2009, the number of cancer deaths increased among white and black Americans primarily because of an aging white population and a growing black population. Overall, age-standardized cancer death rates (risk) declined in all groups. From 2007 to 2020, rates are predicted to continue to decrease while counts of deaths are predicted to increase among men (15%) and stabilize among women (increase <10%). Declining death rates are predicted to meet HP2020 targets for cancers of the female breast, lung and bronchus, cervix and uterus, colon and rectum, oral cavity and pharynx, and prostate, but not for melanoma.

**Conclusion:**

Cancer deaths among women overall are predicted to increase by less than 10%, because of, in part, declines in breast, cervical, and colorectal cancer deaths among white women. Increased efforts to promote cancer prevention and improve survival are needed to counter the impact of a growing and aging population on the cancer burden and to meet melanoma target death rates.

## Introduction

In the United States, the age-standardized cancer death rate began declining in the early 1990s, largely because of declines in deaths from lung and prostate cancer in men, breast cancer in women, and colorectal cancer in both sexes (1). The age-standardized death rate approximates the population’s risk of dying from cancer and is used to compare risk of death between populations or over time within a population. A decline in the death rate means that the overall risk of dying from cancer in the population has decreased. However, age-standardized rates do not convey the full extent of the cancer burden, as they effectively remove the influence of demographic changes in the population. During this time, the observed number of cancer deaths has continued to increase (2).

The number of cancer deaths is a function of the population’s risk of dying from cancer and the population’s age structure and size. The observed increase in the number of cancer deaths reflects the increased risk of dying from cancer with age, and during the past several decades, the US population has grown, particularly in the older age groups (3). These demographic trends and increasing cancer burden are forecast to continue as the cohort born following World War II enters the age groups most at risk of dying from cancer (4).

In 1971, the US Congress passed the National Cancer Act, which signaled a national effort against cancer (5) and led to the establishment of the Surveillance, Epidemiology, and End Results (SEER) Program in 5 states and 4 metropolitan areas (1). Since then, cancer registries (2) and cancer control programs (6) have been established in all states. More recently, the US Department of Health and Human Services (HHS) issued Healthy People 2020 (HP2020) (7), which included several objectives for reducing cancer mortality. Each objective has a baseline measure and a target to be achieved by 2020. Most of the cancer mortality objectives include a 10% reduction in the age-standardized death rate from 2007 (baseline) to 2020. The colorectal cancer target calls for a 15% reduction in death rates.

Trends in population risk, size, and age structure have been used to predict the future of cancer mortality in other countries (8). To determine whether HP2020 cancer mortality targets are likely to be met, we used mortality data and population estimates and projections to assess the contribution of changes in population risk, growth, and aging on cancer deaths from 1975 to 2020 for all cancer sites and the top 23 cancers by sex and race.

## Methods

Mortality data were from the Centers for Disease Control and Prevention (CDC)’s National Vital Statistics System (9). Cause of death information was coded based on the *International Classification of Diseases* (*ICD*) versions in use at the time of death, and cause of death recodes were applied to accommodate consistency over time (2). For these analyses, we selected malignant neoplasms (C00–97). We used population estimates for 1975 through 2009 available from the SEER Program and obtained population projections for 2010 through 2020 from the US Census Bureau’s Population Projections Program (10).

**Figure 1:**
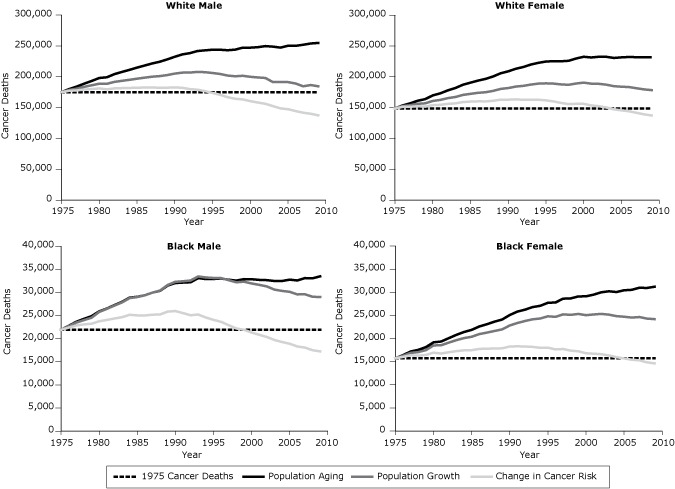
Trends in deaths from all cancers combined attributed to population risk (including diagnostic and treatment practices), growth, and aging (1975–2009), by sex and race (white, black). **Table:** 

To estimate the relative contribution to changes in the total number of new cancer deaths each year that can be attributed to changes in population risk (including changes in diagnosis and treatment practices) and demographic changes related to population size and age structure, we generated 3 sets of data by sex and race (white and black) for each year (1976–2009) based on a method first published in the 1999 Canadian Cancer Statistics report (11). The dotted line in Figure 1 represents the number of deaths from cancer that occurred in 1975. The lowest solid line represents the number of cancer deaths that would have occurred each year if the population size and age structure had remained the same as they were in 1975. This line is similar to the age-standardized death rate and reflects the impact of changes in population risk including changes in diagnosis and treatment practices. The middle line represents the number of deaths that would have occurred if the age structure of the population had remained the same as it was in 1975. This line reflects the impact of changes in risk and population growth. The top line represents the number of deaths that actually occurred and thus reflects the combined impact of changes in population risk, growth, and aging. The yearly difference between each set of death counts denotes the relative contribution to the overall change in the number of deaths since 1975 attributable to population risk, growth, and aging, respectively. A decline in risk during this time results in negative death counts as fewer deaths are attributed to risk compared with the baseline year.

Year of Diagnosis	1975 Cancer Deaths	Population Aging	Population Growth	Population Risk
1975 Cancer Deaths in White Males				
1975	175,299	175,299	175,299	175,299
1976	175,299	180,269	178,746	177,352
1977	175,299	184,261	180,914	178,052
1978	175,299	189,013	183,867	179,411
1979	175,299	193,447	186,412	180,253
1980	175,299	198,189	188,928	181,293
1981	175,299	199,373	188,719	179,733
1982	175,299	204,473	191,831	181,387
1983	175,299	208,060	193,615	181,751
1984	175,299	211,373	195,255	182,056
1985	175,299	215,088	197,098	182,502
1986	175,299	218,383	198,756	182,669
1987	175,299	221,766	200,276	182,777
1988	175,299	224,520	201,280	182,393
1989	175,299	228,317	202,921	182,517
1990	175,299	232,617	205,291	182,901
1991	175,299	236,430	206,933	182,283
1992	175,299	238,504	206,996	180,176
1993	175,299	241,940	208,120	179,168
1994	175,299	243,012	207,603	176,887
1995	175,299	244,026	206,278	173,972
1996	175,299	243,970	204,708	170,958
1997	175,299	243,212	202,090	167,047
1998	175,299	244,124	200,983	164,462
1999	175,299	247,392	201,753	163,476
2000	175,299	247,396	200,120	160,668
2001	175,299	248,142	199,105	158,397
2002	175,299	249,864	198,171	156,349
2003	175,299	249,045	191,520	152,627
2004	175,299	247,446	191,700	148,868
2005	175,299	250,475	191,520	147,529
2006	175,299	250,318	189,252	144,548
2007	175,299	252,047	184,752	141,943
2008	175,299	254,119	186,712	140,184
2009	175,299	255,040	184,752	137,686
1975 Cancer Deaths in White Females				
1975	148,711	148,711	148,711	148,711
1976	148,711	153,688	152,569	151,377
1977	148,711	157,052	153,985	151,478
1978	148,711	161,273	156,378	152,431
1979	148,711	163,890	157,147	151,760
1980	148,711	169,970	160,982	154,197
1981	148,711	173,307	162,654	154,670
1982	148,711	178,167	165,506	156,285
1983	148,711	181,950	167,359	156,996
1984	148,711	187,266	170,715	159,171
1985	148,711	190,649	172,523	159,860
1986	148,711	193,967	174,107	160,301
1987	148,711	196,727	175,124	160,250
1988	148,711	200,633	177,296	161,242
1989	148,711	205,867	180,475	163,099
1990	148,711	208,986	181,889	163,026
1991	148,711	213,108	184,329	163,562
1992	148,711	216,036	185,438	162,893
1993	148,711	219,997	187,521	163,116
1994	148,711	222,817	189,061	162,963
1995	148,711	224,905	189,505	161,927
1996	148,711	225,471	189,125	160,231
1997	148,711	225,336	187,794	157,703
1998	148,711	226,051	187,187	155,854
1999	148,711	229,839	189,280	156,312
2000	148,711	232,608	190,664	156,269
2001	148,711	231,505	188,998	153,759
2002	148,711	232,613	188,708	152,445
2003	148,711	232,500	187,329	150,307
2004	148,711	230,682	184,914	147,380
2005	148,711	231,652	184,070	145,722
2006	148,711	232,251	183,502	144,253
2007	148,711	231,888	181,551	141,649
2008	148,711	231,767	179,705	139,241
2009	148,711	231,947	178,483	137,427
1975 Cancer Deaths in Black Males				
1975	21,884	21,884	21,884	21,884
1976	21,884	22,748	22,673	22,358
1977	21,884	23,616	23,448	22,829
1978	21,884	24,249	23,987	23,055
1979	21,884	24,827	24,490	23,196
1980	21,884	25,855	25,750	23,725
1981	21,884	26,470	26,405	24,004
1982	21,884	27,217	27,127	24,330
1983	21,884	27,927	27,803	24,613
1984	21,884	28,848	28,739	25,128
1985	21,884	29,022	28,964	25,005
1986	21,884	29,360	29,343	24,994
1987	21,884	29,921	29,927	25,157
1988	21,884	30,319	30,379	25,196
1989	21,884	31,450	31,582	25,825
1990	21,884	31,993	32,261	25,937
1991	21,884	32,089	32,386	25,498
1992	21,884	32,156	32,538	25,035
1993	21,884	33,069	33,423	25,188
1994	21,884	32,858	33,205	24,548
1995	21,884	32,878	33,101	24,037
1996	21,884	32,973	33,066	23,608
1997	21,884	32,717	32,607	22,876
1998	21,884	32,521	32,164	22,181
1999	21,884	32,849	32,286	21,892
2000	21,884	32,815	31,906	21,292
2001	21,884	32,676	31,603	20,808
2002	21,884	32,625	31,272	20,341
2003	21,884	32,442	30,575	19,682
2004	21,884	32,441	30,299	19,252
2005	21,884	32,724	30,094	18,881
2006	21,884	32,555	29,541	18,284
2007	21,884	33,067	29,564	18,041
2008	21,884	33,018	29,031	17,477
2009	21,884	33,441	28,968	17,218
1975 Cancer Deaths in Black Females				
1975	15,747	15,747	15,747	15,747
1976	15,747	16,408	16,285	16,034
1977	15,747	17,197	16,897	16,385
1978	15,747	17,559	17,075	16,307
1979	15,747	18,147	17,499	16,440
1980	15,747	19,173	18,504	16,940
1981	15,747	19,346	18,552	16,756
1982	15,747	20,086	19,118	17,030
1983	15,747	20,803	19,648	17,277
1984	15,747	21,407	20,095	17,455
1985	15,747	21,874	20,381	17,484
1986	15,747	22,610	20,955	17,750
1987	15,747	23,093	21,286	17,797
1988	15,747	23,644	21,634	17,845
1989	15,747	24,114	21,985	17,885
1990	15,747	25,082	22,824	18,271
1991	15,747	25,826	23,356	18,316
1992	15,747	26,245	23,783	18,269
1993	15,747	26,796	24,154	18,189
1994	15,747	27,077	24,326	17,979
1995	15,747	27,723	24,806	18,021
1996	15,747	27,793	24,695	17,649
1997	15,747	28,616	25,223	17,729
1998	15,747	28,672	25,124	17,377
1999	15,747	29,101	25,319	17,243
2000	15,747	29,127	25,049	16,819
2001	15,747	29,490	25,198	16,720
2002	15,747	29,989	25,330	16,622
2003	15,747	30,218	25,163	16,344
2004	15,747	30,056	24,846	15,966
2005	15,747	30,437	24,716	15,708
2006	15,747	30,526	24,530	15,411
2007	15,747	30,978	24,641	15,288
2008	15,747	30,934	24,309	14,862
2009	15,747	31,204	24,195	14,624

To project age-standardized death rates and counts for 2010–2020, we used Nordpred software (12), which uses an age-period-cohort regression model with data aggregated into six 5-year calendar periods (1980–2009) and fifteen 5-year age groups (15–19, …, 80–84, ≥85). We fit a separate model for each of the current top 23 cancer causes of death and all other cancer deaths combined by sex and race (all, white, and black): R*_ap_* = (A_a_ + D × p + P_p_ + C_c_)^5 ^where *R_ap_* is the death rate in age group a in calendar period p, A_a_ is the age component for age group a, D is the drift parameter (the common linear effect of both calendar period and birth cohort), P_p_ is the nonlinear period component of period p, and C_c_ is the nonlinear cohort component of cohort. To offset exponential increases or decreases in death rates, the models used the Power-5 link function. Assuming that trends are not likely to continue indefinitely, we reduced the drift component D by 25% and 50% in the second and third 5-year periods, respectively. Both these modifications have been shown empirically to improve predictions. Nordpred uses a goodness-of-fit test to determine the optimum number of calendar periods (4–6 candidate periods are sufficient) to include in the model (12). Significant curvature over time was determined by comparing a model with and without a second order term for calendar period (p), using a χ^2^ test for the difference in residual sum of squares between the models. If there was significant curvature, the slope from the last 10 years was projected; if not, the average linear trend (D) was used. Visual data inspection determined the starting age for each cancer site by sex and race such that each age group contained no fewer than 10 deaths. We calculated age-standardized death rates (per 100,000) using the US 2000 standard population weights (13). Predicted death counts were obtained by applying predicted age-specific death rates to the 2010 through 2020 population projections. We apportioned cancer death counts by changes in population risk and in demographics (population size and age structure combined) (14). We considered a change of 10% or more in age-adjusted rates or death counts as an increase or decrease; otherwise, we considered rates and death counts to be stable.

## Results

From 1975 through 2009, the number of cancer deaths increased 45.5% among white males, 56.0% among white females, 52.8% among black males, and 98.2% among black females (Table 1, Figure 1). For each sex and race group, the number of cancer deaths attributed to risk decreased, whereas the number of cancer deaths attributed to population growth and aging increased as follows: white males (−21.5% risk, 26.8% growth, 40.1% aging); white females (−7.6% risk, 27.6% growth, 36.0% aging); black males (−21.3% risk, 53.7% growth, 20.4% aging); and black females (−7.1% risk, 60.8% growth, 44.5% aging) (Table 1).

**Table 1:** Observed Cancer Deaths (All Sites Combined) and Percentage Change From Population Risk, Growth, and Aging by Sex and Race, 1975–2009

Death Year	Male				Female			
	Total	Risk	Growth	Aging	Total	Risk	Growth	Aging
**White**								
1975	175,299	NA	NA	NA	148,711	NA	NA	NA
1980	198,189	5,994	7,635	9,261	169,970	5,486	6,781	8,993
1990	232,617	7,602	22,391	27,325	208,986	14,315	18,864	27,096
2000	247,396	−14,631	39,464	47,265	232,608	7,558	34,396	41,943
2009	255,040	−37,613	47,063	70,292	231,947	−11,284	41,053	53,467
% Change 1975–2009	45.5	−21.5	26.8	40.1	56.0	−7.6	27.6	36.0
**Black**								
1975	21,884	NA	NA	NA	15,747	NA	NA	NA
1980	25,855	1,841	2,024	106	19,173	1,193	1,562	671
1990	31,993	4,053	6,325	−269	25,082	2,524	4,554	2,258
2000	32,815	−592	10,615	908	29,127	1,072	8,228	4,080
2009	33,441	−4,666	11,749	4,474	31,204	−1,123	9,573	7,007
% Change 1975–2009	52.8	−21.3	53.7	20.4	98.2	−7.1	60.8	44.5

Abbreviations: NA, not applicable.

**Figure 2:**
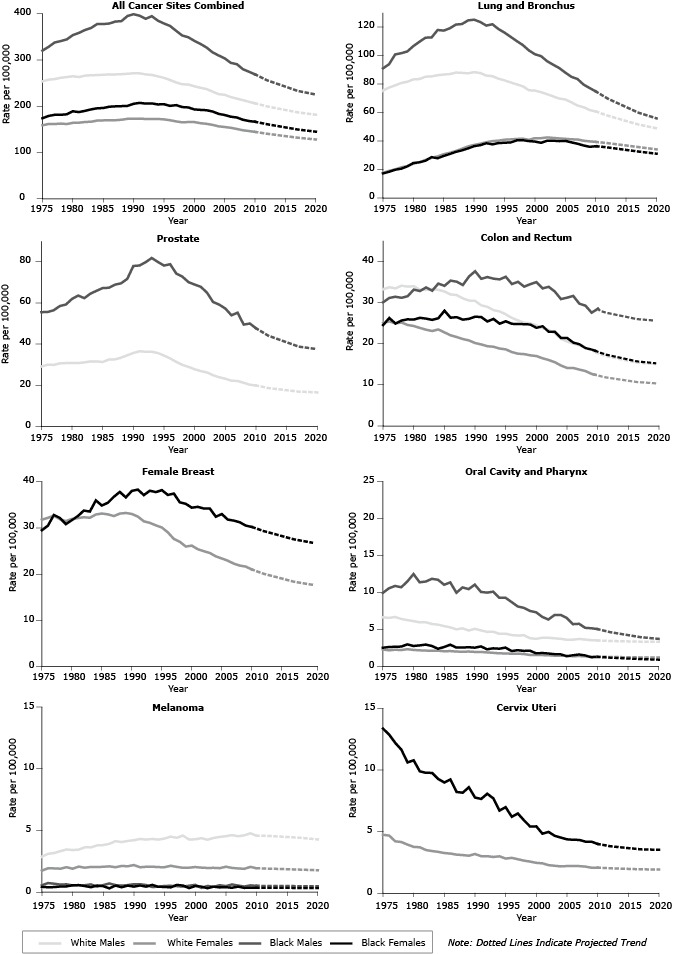
Trends in observed and predicted age-adjusted death rates for all sites combined and for the 7 site-specific cancers included in the Healthy People 2020 cancer mortality objectives by sex and race, 1975–2020. **Table:** 

Figure 2 shows 1975 through 2009 (observed) and 2010 through 2020 (predicted) age-standardized cancer death rates for all sites combined and for the 7 site-specific cancers included in the cancer mortality objectives for Healthy People 2020. (Additional information can be found at http://www.cdc.gov/cancer/dcpc/research/articles/cancer_2020_figures.htm.) Table 2 shows predicted 2020 deaths by race (all races combined, white, black), sex, and cancer site, with the percentage change in deaths from 2007 (baseline) and 2020 apportioned because of changes in risk and demographics (population growth and aging combined). By 2020, we predict cancer deaths to increase 15.2% among men (−23.0% attributable to risk, 38.2% attributable to demographics) and begin to stabilize (8.1%) among women (−19.5% attributable to risk, 27.6% attributable to demographics). Results varied by cancer site, sex, and race. We predict that cancer risk will decrease for 13 of 19 cancers among men and 15 of 21 cancers in women, while the number of deaths will increase for 11 of 19 cancer sites among men and 9 of 21 cancer sites among women, in large part because of the demographic component. We predict that risk of death will increase for cancers of the corpus and uterus among black women, liver and intrahepatic bile duct among both men and women (both races), and for thyroid cancer in women (both races). We predict that the number of deaths for colorectal cancer and non-Hodgkin’s lymphoma will decrease among white women, for esophageal cancer among black men and women, Hodgkin’s lymphoma among white men and women, laryngeal cancer among black men, and cancers of the oral cavity and pharynx among black women.

Year	White Male	White Female	Black Male	Black Female
All Cancer Sites Combined				
1975	254.00	158.99	320.45	174.18
1976	257.50	161.69	328.45	179.10
1977	258.78	161.61	337.95	181.60
1978	261.64	162.54	340.91	181.53
1979	263.24	161.69	344.67	182.73
1980	265.02	164.17	353.91	189.24
1981	263.26	164.55	358.65	187.47
1982	266.28	166.11	364.86	190.17
1983	267.22	166.69	369.32	193.24
1984	267.55	169.02	377.93	195.67
1985	268.24	169.58	377.82	196.31
1986	268.83	170.01	378.92	199.05
1987	268.96	169.87	383.24	199.91
1988	269.46	170.94	384.17	200.36
1989	270.25	172.88	395.28	200.87
1990	271.48	172.89	399.14	205.38
1991	271.19	173.41	395.85	207.39
1992	268.91	172.58	389.20	205.80
1993	267.89	172.82	394.76	206.29
1994	264.73	172.62	385.20	204.06
1995	261.46	171.73	379.02	204.53
1996	256.83	169.85	373.70	200.94
1997	251.58	167.35	363.18	202.53
1998	247.62	165.38	353.13	198.38
1999	247.14	165.92	349.23	197.54
2000	243.31	165.94	341.48	193.26
2001	240.03	163.52	334.93	192.18
2002	237.25	162.14	327.14	191.51
2003	231.79	159.81	316.59	188.81
2004	226.30	156.68	309.94	183.64
2005	224.75	155.18	303.63	181.13
2006	219.98	153.59	293.91	177.57
2007	216.61	150.84	291.00	175.78
2008	213.29	148.23	280.09	170.95
2009	209.78	146.46	274.73	168.17
2010	206.52	144.69	268.96	166.51
2011	203.12	142.66	262.53	163.83
2012	199.72	140.63	256.09	161.14
2013	197.16	138.94	251.54	158.88
2014	194.59	137.24	246.99	156.63
2015	192.02	135.54	242.45	154.37
2017	186.88	132.15	233.35	149.85
2018	185.20	130.87	230.68	148.28
2019	183.53	129.58	228.00	146.71
2020	181.85	128.30	225.33	145.15
Cervix Uteri				
1975	—	4.75	—	13.35
1976	—	4.68	—	12.86
1977	—	4.22	—	12.19
1978	—	4.15	—	11.64
1979	—	3.95	—	10.61
1980	—	3.77	—	10.78
1981	—	3.74	—	9.89
1982	—	3.51	—	9.77
1983	—	3.43	—	9.76
1984	—	3.36	—	9.27
1985	—	3.27	—	8.98
1986	—	3.22	—	9.23
1987	—	3.13	—	8.22
1988	—	3.10	—	8.15
1989	—	3.05	—	8.60
1990	—	3.19	—	7.77
1991	—	3.01	—	7.65
1992	—	3.01	—	8.07
1993	—	2.95	—	7.69
1994	—	3.00	—	6.71
1995	—	2.82	—	6.98
1996	—	2.87	—	6.20
1997	—	2.76	—	6.47
1998	—	2.65	—	5.91
1999	—	2.57	—	5.41
2000	—	2.47	—	5.43
2001	—	2.43	—	4.83
2002	—	2.28	—	4.98
2003	—	2.23	—	4.66
2004	—	2.18	—	4.52
2005	—	2.21	—	4.38
2006	—	2.21	—	4.34
2007	—	2.20	—	4.33
2008	—	2.17	—	4.19
2009	—	2.07	—	4.19
2010	—	2.09	—	4.01
2011	—	2.06	—	3.91
2012	—	2.03	—	3.82
2013	—	2.01	—	3.77
2014	—	2.00	—	3.72
2015	—	1.98	—	3.67
2016	—	1.96	—	3.62
2017	—	1.95	—	3.57
2018	—	1.94	—	3.55
2019	—	1.94	—	3.54
2020	—	1.93	—	3.53
Colon and Rectum				
1975	33.24	25.06	30.09	24.55
1976	33.76	25.45	31.16	26.26
1977	33.49	24.97	31.44	24.93
1978	34.12	25.17	31.20	25.66
1979	33.92	24.57	31.57	25.96
1980	33.99	24.32	33.18	25.89
1981	33.16	23.85	32.83	26.32
1982	33.11	23.42	33.71	26.12
1983	33.32	23.11	32.90	25.82
1984	33.12	23.49	34.65	26.22
1985	32.74	22.77	34.10	28.04
1986	32.01	22.07	35.36	26.35
1987	31.85	21.63	35.10	26.48
1988	31.04	21.16	34.29	25.88
1989	30.52	20.82	36.37	26.07
1990	30.43	20.18	37.64	26.59
1991	29.35	19.85	35.80	26.50
1992	28.99	19.42	36.23	25.43
1993	28.18	19.28	35.84	26.04
1994	27.89	18.84	35.66	24.88
1995	27.16	18.64	36.30	25.47
1996	26.29	17.98	34.52	24.86
1997	25.68	17.59	35.07	24.80
1998	25.13	17.48	33.90	24.78
1999	24.99	17.20	34.50	24.70
2000	24.45	17.01	35.01	23.89
2001	23.80	16.48	33.47	24.25
2002	23.34	16.06	33.86	22.95
2003	22.57	15.51	32.71	22.91
2004	21.23	14.72	30.87	21.41
2005	20.56	14.13	31.21	21.42
2006	20.02	14.12	31.65	20.29
2007	19.64	13.73	29.76	19.88
2008	18.98	13.36	29.28	18.97
2009	18.59	12.68	27.57	18.59
2010	18.00	12.53	28.48	18.33
2011	17.49	12.17	28.03	17.84
2012	16.99	11.82	27.57	17.36
2013	16.67	11.60	27.26	17.03
2014	16.36	11.37	26.95	16.70
2015	16.05	11.15	26.64	16.38
2016	15.74	10.93	26.32	16.05
2017	15.43	10.71	26.01	15.73
2018	15.31	10.60	25.89	15.57
2019	15.18	10.50	25.77	15.42
2020	15.06	10.40	25.65	15.27
Female Breast				
1975	—	31.79	—	29.49
1976	—	32.17	—	30.47
1977	—	32.67	—	32.80
1978	—	31.90	—	32.14
1979	—	31.48	—	30.82
1980	—	31.93	—	31.68
1981	—	32.12	—	32.55
1982	—	32.31	—	33.75
1983	—	32.20	—	33.53
1984	—	32.90	—	35.94
1985	—	33.11	—	34.85
1986	—	32.93	—	35.44
1987	—	32.57	—	36.73
1988	—	33.09	—	37.78
1989	—	33.23	—	36.61
1990	—	33.02	—	38.00
1991	—	32.45	—	38.28
1992	—	31.43	—	37.09
1993	—	31.09	—	38.04
1994	—	30.56	—	37.74
1995	—	30.09	—	38.18
1996	—	29.05	—	37.13
1997	—	27.62	—	37.43
1998	—	27.01	—	35.53
1999	—	25.98	—	35.21
2000	—	26.17	—	34.38
2001	—	25.40	—	34.56
2002	—	24.97	—	34.20
2003	—	24.58	—	34.19
2004	—	23.85	—	32.42
2005	—	23.39	—	32.99
2006	—	22.92	—	31.80
2007	—	22.28	—	31.58
2008	—	21.85	—	31.22
2009	—	21.65	—	30.49
2010	—	21.01	—	30.22
2011	—	20.55	—	29.77
2012	—	20.08	—	29.31
2013	—	19.74	—	28.96
2014	—	19.40	—	28.60
2015	—	19.06	—	28.25
2016	—	18.72	—	27.89
2017	—	18.38	—	27.54
2018	—	18.16	—	27.30
2019	—	17.94	—	27.06
2020	—	17.73	—	26.82
Lung and Bronchus				
1975	75.47	17.67	91.01	17.34
1976	77.55	19.10	93.96	18.51
1977	79.09	20.09	100.80	19.91
1978	80.76	21.58	101.65	20.66
1979	81.64	22.42	102.89	22.24
1980	83.27	24.22	106.67	24.58
1981	83.52	25.10	109.71	25.10
1982	85.18	26.72	112.55	26.30
1983	85.40	28.28	112.76	28.73
1984	86.24	29.27	117.98	27.98
1985	86.69	30.81	117.50	29.64
1986	87.03	31.76	119.18	31.00
1987	88.09	33.04	121.77	32.48
1988	87.86	34.57	122.15	33.54
1989	87.57	36.27	124.72	34.87
1990	88.40	37.31	125.17	36.48
1991	87.76	38.12	123.58	37.16
1992	85.98	39.21	121.06	38.55
1993	85.52	39.97	121.98	37.72
1994	83.78	40.30	118.63	38.61
1995	82.62	40.97	116.08	38.84
1996	81.15	41.22	113.18	39.08
1997	79.83	41.52	110.21	40.53
1998	78.47	41.69	107.40	40.67
1999	75.67	40.86	103.61	40.00
2000	75.41	42.08	100.96	39.66
2001	74.33	42.02	99.58	38.81
2002	72.88	42.59	96.04	40.22
2003	71.34	42.22	93.59	40.25
2004	69.81	41.89	91.25	39.95
2005	69.22	41.59	88.04	40.15
2006	67.23	41.24	85.09	39.09
2007	64.97	41.15	83.58	38.16
2008	63.75	40.23	79.64	36.93
2009	61.77	39.84	77.22	35.99
2010	60.81	39.44	74.87	36.52
2011	59.31	38.99	72.31	36.02
2012	57.81	38.54	69.75	35.52
2013	56.57	38.00	67.79	34.95
2014	55.34	37.46	65.83	34.38
2015	54.11	36.91	63.87	33.81
2016	52.88	36.37	61.92	33.24
2017	51.65	35.83	59.96	32.67
2018	50.75	35.28	58.62	32.16
2019	49.86	34.73	57.29	31.65
2020	48.96	34.18	55.95	31.13
Melanoma				
1975	2.88	1.74	0.56	0.44
1976	3.10	1.94	0.74	0.39
1977	3.17	1.93	0.69	0.41
1978	3.32	1.90	0.61	0.46
1979	3.46	2.02	0.64	0.47
1980	3.41	1.90	0.55	0.56
1981	3.45	2.07	0.56	0.57
1982	3.64	1.99	0.56	0.49
1983	3.63	2.04	0.63	0.40
1984	3.79	2.03	0.45	0.54
1985	3.82	2.06	0.58	0.49
1986	3.92	2.09	0.70	0.31
1987	4.13	2.02	0.57	0.54
1988	4.07	2.13	0.53	0.40
1989	4.15	2.10	0.57	0.53
1990	4.21	2.20	0.65	0.45
1991	4.31	2.02	0.65	0.54
1992	4.27	2.06	0.57	0.44
1993	4.31	2.06	0.42	0.59
1994	4.26	2.03	0.44	0.43
1995	4.35	2.02	0.51	0.42
1996	4.50	2.14	0.51	0.38
1997	4.41	2.05	0.49	0.58
1998	4.59	1.98	0.45	0.54
1999	4.27	1.98	0.52	0.33
2000	4.28	2.03	0.60	0.50
2001	4.38	1.99	0.35	0.45
2002	4.25	1.96	0.50	0.32
2003	4.39	1.97	0.44	0.45
2004	4.48	1.94	0.57	0.39
2005	4.54	2.06	0.49	0.41
2006	4.63	1.97	0.63	0.35
2007	4.53	1.92	0.54	0.45
2008	4.61	1.89	0.46	0.35
2009	4.77	2.05	0.55	0.36
2010	4.58	1.94	0.51	0.37
2011	4.57	1.92	0.51	0.37
2012	4.56	1.91	0.50	0.36
2013	4.53	1.89	0.50	0.36
2014	4.50	1.88	0.50	0.35
2015	4.47	1.86	0.49	0.35
2016	4.44	1.85	0.49	0.35
2017	4.41	1.83	0.49	0.34
2018	4.37	1.81	0.48	0.34
2019	4.32	1.79	0.48	0.34
2020	4.27	1.78	0.48	0.34
Oral Cavity and Pharynx				
1975	6.64	2.29	9.93	2.52
1976	6.55	2.17	10.55	2.62
1977	6.67	2.25	10.87	2.65
1978	6.41	2.21	10.69	2.68
1979	6.25	2.32	11.47	2.97
1980	6.12	2.22	12.48	2.76
1981	5.96	2.17	11.36	2.84
1982	5.96	2.13	11.46	2.94
1983	5.73	2.10	11.83	2.76
1984	5.64	2.12	11.70	2.38
1985	5.44	2.05	11.03	2.62
1986	5.27	2.07	11.35	2.93
1987	5.01	2.00	9.95	2.54
1988	5.14	1.98	10.67	2.55
1989	4.84	2.00	10.44	2.59
1990	5.07	1.96	11.05	2.52
1991	4.87	1.95	10.06	2.70
1992	4.66	1.89	9.97	2.31
1993	4.68	1.83	10.09	2.44
1994	4.41	1.77	9.26	2.39
1995	4.42	1.75	9.28	2.54
1996	4.24	1.71	8.69	2.07
1997	4.17	1.70	8.10	2.19
1998	4.20	1.68	7.91	2.09
1999	3.80	1.54	7.49	2.12
2000	3.72	1.55	7.31	1.76
2001	3.87	1.52	6.69	1.81
2002	3.87	1.50	6.33	1.73
2003	3.80	1.45	6.95	1.65
2004	3.72	1.46	6.95	1.63
2005	3.60	1.43	6.54	1.38
2006	3.61	1.37	5.70	1.50
2007	3.71	1.39	5.76	1.60
2008	3.62	1.37	5.21	1.47
2009	3.54	1.31	5.15	1.23
2010	3.51	1.32	5.04	1.29
2011	3.47	1.28	4.84	1.24
2012	3.44	1.27	4.64	1.19
2013	3.42	1.25	4.50	1.15
2014	3.40	1.24	4.36	1.11
2015	3.38	1.23	4.23	1.07
2016	3.36	1.21	4.09	1.03
2017	3.35	1.21	3.95	1.00
2018	3.34	1.20	3.87	0.97
2019	3.33	1.19	3.80	0.94
2020	3.33	1.28	3.72	0.91
Prostate				
1975	29.06	—	55.52	—
1976	29.93	—	55.60	—
1977	29.90	—	56.44	—
1978	30.62	—	58.49	—
1979	30.77	—	59.24	—
1980	30.77	—	61.99	—
1981	30.76	—	63.61	—
1982	31.09	—	62.36	
1983	31.57	—	64.55	—
1984	31.58	—	65.93	—
1985	31.28	—	67.26	—
1986	32.46	—	67.39	—
1987	32.57	—	68.88	—
1988	33.38	—	69.57	—
1989	34.54	—	71.71	—
1990	35.67	—	77.98	—
1991	36.50	—	78.24	—
1992	36.29	—	79.84	—
1993	36.26	—	81.86	—
1994	35.64	—	79.96	—
1995	34.41	—	78.20	—
1996	33.00	—	78.84	—
1997	31.35	—	74.30	—
1998	29.89	—	72.77	—
1999	28.91	—	70.13	—
2000	27.75	—	68.93	—
2001	26.87	—	67.86	—
2002	26.20	—	65.04	—
2003	24.85	—	60.48	—
2004	23.86	—	59.20	—
2005	23.15	—	57.29	—
2006	22.17	—	54.01	—
2007	22.05	—	55.27	—
2008	21.17	—	49.49	—
2009	20.22	—	50.00	—
2010	19.86	—	47.69	—
2011	19.24	—	45.89	—
2012	18.62	—	44.09	—
2013	18.27	—	43.02	—
2014	17.92	—	41.95	—
2015	17.57	—	40.88	—
2016	17.22	—	39.81	—
2017	16.86	—	38.74	—
2018	16.74	—	38.35	—
2019	16.61	—	37.95	—
2020	16.49	—	37.55	—

Abbreviation: —, not applicable.

**Table 2:** Predicted (2020) Deaths and Percentage Change From 2007 to 2020 by Risk and Demographics (Population Growth and Aging Combined) for All Cancer Sites Combined and for the Top 23 Cancers by Race and Sex

Cancer Site	All Races							White					Black					
	2020, No.		% Change From 2007 to 2020					2020, No.	% Change From 2007 to 2020				2020, No.	% Change From 2007 to 2020				
			Overall		Attributable to				Overall		Attributable to			Overall		Attributable to		
					Risk	Dem					Risk	Dem				Risk		Dem
**Males**																		
All cancer sites combined	337,280			15.2	−23.0	38.2		285,800	13.4		−21.4	34.8	38,248	15.7		−32.7		48.3
Brain and other CNS	8,752			19.6	−11.5	31.1		7,920	17.2		−10.0	27.2	530	31.2		−9.1		40.3
Colon and rectum	29,244			8.3	−29.0	37.3		23,568	3.3		−30.7	33.9	4,375	27.9		−19.5		47.4
Esophagus	14,313			33.1	−4.9	38.0		13,010	37.1		2.9	34.1	899	−14.8		−65.3		50.6
Hodgkin lymphoma	680			−3.7	−29.5	25.8		553	−11.0		−33.9	22.9	72	−2.5		−32.1		29.6
Kidney and renal pelvis	9,788			22.9	−14.4	37.3		7,932	13.4		−20.4	33.7	1,032	38.7		−7.3		46.0
Larynx	3,047			5.4	−31.9	37.3		2,512	10.0		−23.6	33.7	468	−14.5		−63.6		49.1
Leukemia	15,006			20.7	−15.5	36.2		13,278	18.7		−14.4	33.1	1,224	27.9		−14.7		42.6
Liver and IBD	20,757			83.0	49.8	33.2		16,216	82.5		52.8	29.7	2,983	87.4		42.8		44.6
Lung and bronchus	91,592			3.7	−37.1	40.8		78,588	2.8		−34.4	37.2	9,817	−0.2		−51.6		51.4
Melanoma	6,728			22.2	−11.4	33.6		6,546	21.0		−8.6	29.6	81	36.7		−4.5		41.2
Myeloma	6,831			17.6	−22.0	39.5		5,403	12.8		−23.6	36.4	1,157	32.5		−16.1		48.7
Non-Hodgkin lymphoma	10,662			−3.1	−39.9	36.8		9,369	−5.8		−39.5	33.7	893	19.2		−23.9		43.1
Oral cavity and pharynx	6,216			12.8	−22.4	35.2		5,389	18.3		−13.4	31.7	698	−9.2		−55.8		46.6
Pancreas	24,339			42.1	3.6	38.4		20,674	39.9		5.1	34.8	2,541	34.2		−14.3		48.6
Prostate	30,914			6.3	−33.9	40.2		24,778	4.7		−32.4	37.1	5,285	7.7		−41.6		49.3
Stomach	6,857			1.5	−35.3	36.8		4,951	−2.9		−36.4	33.5	1,264	6.0		−40.5		46.5
Testis	356			9.1	−3.4	12.4		315	6.9		−1.5	8.3	—	—		—		—
Thyroid	967			39.3	1.0	38.3		841	39.2		4.8	34.4	—	—		—		—
Urinary bladder	13,113			36.0	−3.3	39.3		11,946	33.5		−2.8	36.2	887	56.9		8.9		48.0
**Females**																		
All cancer sites combined	291,923	8.1			−19.5		27.6	242,196		4.4	−18.4	22.8	36,392		17.5	−24.9	42.3	
Brain and other CNS	6,771	14.4			−12.0		26.4	6,050		11.7	−10.1	21.7	438		13.2	−24.1	37.3	
Cervix uteri	4,085	1.6			−17.7		19.3	3,032		−0.2	−14.3	14.1	856		6.3	−26.5	32.8	
Colon and rectum	24,887	−5.1			−30.2		25.1	19,834		−10.0	−30.1	20.2	3,802		10.1	−32.9	42.9	
Corpus and uterus, NOS	10,407	39.6			8.7		30.9	7,847		31.6	6.7	25.0	2,092		61.6	10.6	50.9	
Esophagus	3,004	5.7			−23.7		29.4	2,672		12.1	−12.5	24.6	339		−14.9	−58.7	43.9	
Female breast	40,434	−0.4			−25.5		25.0	32,065		−5.3	−25.8	20.6	6,683		15.3	−21.6	36.9	
Hodgkin lymphoma	490	−13.2			−35.3		22.0	424		−15.3	−33.1	17.8	55		−2.5	−33.6	31.1	
Kidney and renal pelvis	5,136	8.4			−19.5		27.9	4,382		5.7	−17.6	23.3	516		8.0	−33.6	41.6	
Larynx	863	15.9			−16.0		31.9	747		20.1	−6.1	26.2	118		5.8	−44.1	49.9	
Leukemia	10,180	7.2			−17.6		24.8	8,825		5.3	−14.9	20.2	923		3.7	−36.2	39.9	
Liver and IBD	8,576	47.8			20.7		27.1	6,853		44.9	22.5	22.4	1,046		55.6	13.8	41.8	
Lung and bronchus	76,679	9.0			−22.7		31.7	65,793		5.8	−21.1	26.8	7,926		19.2	−26.6	45.7	
Melanoma	3,361	13.7			−9.5		23.2	3,195		12.1	−6.1	18.2	82		6.3	−34.3	40.5	
Myeloma	4,811	−5.0			−34.0		29.1	3,611		−8.7	−32.6	24.0	947		−5.0	−51.4	46.3	
Non-Hodgkin lymphoma	7,831	−17.8			−43.2		25.5	6,813		−21.1	−42.1	20.9	641		0.0	−39.9	39.8	
Oral cavity and pharynx	2,615	2.3			−23.5		25.8	2,230		3.0	−18.5	21.5	232		−20.9	−61.0	40.1	
Ovary	16,432	12.4			−16.3		28.7	14,122		7.9	−15.9	23.7	1,398		20.5	−24.0	44.5	
Pancreas	23,117	36.1			7.9		28.2	18,999		32.7	9.4	23.3	2,981		37.0	−7.9	44.9	
Stomach	4,460	−3.7			−27.8		24.1	3,159		−8.5	−27.4	18.9	822		2.1	−39.5	41.5	
Thyroid	1,297	49.4			21.2		28.2	1,006		41.5	18.8	22.7	172		82.5	34.9	47.7	
Urinary bladder	4,961	18.1			−6.1		24.2	4,289		16.9	−3.0	19.9	544		16.1	−25.0	41.1	

Abbreviations: —, Data not shown because numbers were too small for analysis; Dem, demographics.

Table 3 shows observed (2007), predicted (2020), and HP2020 target age-standardized death rates per 100,000 population, percent change from 2007 to 2020, and the year in which we predict death rates to meet HP2020 targets by cancer site. We predict declining death rates from 2007 to 2020 for the following cancers: all sites combined (15.6%), lung and bronchus (21.3%), female breast (19.6%), cervix uteri (12.5%), colon and rectum (22.5%), oral cavity and pharynx (16.0%), prostate (26.4%), and melanoma of the skin (7.4%). The years in which we project the age-standardized death rate to meet the HP2020 target rates are 2010 (prostate), 2011 (cervix uteri, oral cavity and pharynx), 2012 (female breast), 2013 (lung and bronchus, colon and rectum), and 2015 (all sites combined). We do not predict that melanoma age-standardized death rates will meet the goal of a 10% reduction by 2020.

**Table 3:** Observed (2007) and Predicted (2020) Age-Standardized Death Rates (ASDR) per 100,000 Population and Overall Percentage Change for All Cancer Sites Combined and for the 7 Site-Specific Cancers Included in the Healthy People 2020 Cancer Mortality Objectives

HP2020 Objectives/Cancer Site	2007 Observed ASDR	2020 Predicted ASDR	Percentage Change (2007–2020) ASDR	HP2020 Target ASDR	Year Predicted to Meet HP2020 Target
C-1, All cancer sites combined	179.3	151.4	−15.6	161.4	2015
C-2, lung and bronchus	50.7	39.8	−21.3	45.5	2013
C-3, female breast	23.0	18.5	−19.6	20.7	2012
C-4, cervix uteri	2.4	2.1	−12.5	2.2	2011
C-5, colon and rectum	16.9	13.1	−22.5	14.5	2013
C-6, oral cavity and pharynx	2.5	2.1	−16.0	2.3	2011
C-7, prostate	24.2	17.8	−26.4	21.8	2010
C-8, melanoma	2.7	2.5	−7.4	2.4	—

Abbreviations: —, will not achieve goal of a 10% reduction by 2020; HP2020, Healthy People 2020.

## Discussion

Age-standardized death rates (population risk) began declining in the early 1990s and are predicted to continue to decline through 2020 for all cancer sites combined and for many of the leading cancers in both men and women. However, we predict that the total number of cancer deaths from 2007 to 2020 will increase more than 10% among men and black women and will begin to stabilize among white women, increasing only 4.4% during this period. Thus, while the overall risk of dying from cancer is declining, the impact of underlying demographic changes in the population will increase the burden of cancer on society and health care systems (15–17).

From 1975 to 2009, the observed number of cancer deaths increased among white Americans primarily because of an aging white population and among black Americans primarily because of a growing black population. Population aging has only recently begun to contribute to the increasing number of cancers deaths among black men. This shift can be explained by inequities in life expectancy. Compared with white Americans, life expectancy among black Americans in general and black men in particular remains lower because of higher death rates from heart disease, cancer, homicide, diabetes, and perinatal conditions (18). The demographic component underlying the increase in the number of cancer deaths is likely to continue into this century. Overall, the US population is expected to increase by 10% from 2010 to 2020; the largest increases are expected in minority populations and in the proportion of people older than 65 (13% to 16%) (3).

To help decrease the burden of cancer in the United States, HP2020 objectives called for a reduction in age-standardized death rates for all cancers combined, melanoma, and cancers of the female breast, cervix uteri, colon and rectum, lung and bronchus, oral cavity and pharynx, and prostate. In 2007, deaths from these cancers comprised the majority of cancer deaths (2). Many of these deaths could be prevented through reduced incidence of cancer, improved survival, or both. We predict that HP2020 target rates will be met first for prostate cancer in 2010 and lastly for all sites combined by 2015. We do not predict that melanoma cancer death rates will reach the HP2020 target rate by 2020. An examination of the most recent mortality data (9) shows that the HP2020 target for prostate cancer was met in 2010 and that the mortality rates for the other HP2020 targets declined as predicted with the exception of oral cavity and pharynx, which may be stabilizing.

An overarching goal of HP2020 is to improve the health of all population subgroups. These projections help inform the potential achievement of this goal. From 2007 to 2020, we predict a reduction of greater than 15% in death rates for colorectal cancer and a reduction of greater than 10% in death rates for cancers of the (female) breast, cervix, lung, oral cavity, and prostate, among white and black Americans. Despite this improvement, we expect that the total overall number of cancer deaths will increase in white men, black men, and black women and will begin to stabilize among white women. This trend reflects the impact of 2 leading cancers: we predict a modest 5.3% decline in the number of breast cancer deaths among white women, compared with a 15.3% increase among black women, and we predict that colorectal cancer deaths will decline by 10% among white women, stabilize among white men, and increase among black men and women.

The stabilization in deaths from lung cancer among men helps measure the success of primary prevention strategies aimed at reducing the incidence of cancer, particularly among highly fatal cancers. Incidence rates for lung cancer have been declining in parallel to a reduction in tobacco use (19). Among black women, the reduction in population risk is predicted to offset only partially the increase in lung cancer deaths attributable to demographic changes, and deaths are predicted to increase nearly 20% by 2020. Deaths from oral cancer are predicted to stabilize or decrease among women and black men during this period.

A reduction in the number of deaths from breast, cervical, and colorectal cancer among white women reflects the success of screening efforts and improved treatment. The reduction in age-standardized colorectal cancer death rates is consistent with a large reduction from screening and smaller reductions from risk factors and improved treatments (20). Access to quality health care, including earlier diagnosis and evidence-based treatments, has resulted in increased survival accompanied by reduced mortality for cancers of the colon and rectum and to a lesser extent, female breast and prostate (21).

Cancer projections can also alert researchers to the impact of changes in population risk before the full extent of the cancer burden manifests. This study identified several cancers in which increasing risk of death is exacerbating demographic trends, including cancers of corpus and uterus in black women, liver and intrahepatic bile duct cancer in both sexes and races, and thyroid cancer in women. Increasing cancer deaths, in part, reflect predicted increases in incidence rates of these cancers (22), possibly the result of a growing obesity epidemic (corpus and uterus cancer) (23), and an epidemic increase in hepatitis infections, particularly among those born from 1945 through 1965 (liver cancer) (24). The increase in deaths from thyroid cancer might be caused by unidentified risk factors operating at the population level and not the result of increased incidence associated with improved detection and access to care (25,26).

Since 1975, melanoma incidence rates have increased among all age groups, whereas death rates have remained unchanged overall and increased in older age groups (27). Melanoma deaths are predicted to increase through 2020 because incidence rates and risk are predicted to continue to increase (22), and death rates and risk are predicted to fall short of the HP2020 target of a 10% reduction from 2007 to 2020. To address the increasing melanoma burden, HHS issued *The Surgeon General’s Call to Action to Prevent Skin Cancer,* which includes strategies to reduce ultraviolet exposure from the sun and indoor tanning, thereby reducing melanoma incidence (28).

The estimates from our study are probably conservative, as the risk component in these models does not account for the potential for advances in primary prevention and treatment (29). Further reductions in cancer mortality, including melanoma, might yet be achievable if HP2020 objectives related to screening and access to health care are met (30).

This study used methods based on age-period cohort models that identify trends in younger birth cohorts and extrapolate these trends to future older cohorts (12,14). Although these methods have been validated in studies using long-term incidence data and are based on the best available information, they are subject to many known limitations. First, the population projections underlying the predicted death rates are themselves forecasts of the population size and age composition based on assumptions regarding future births, deaths, and migration. As such, these projections have the potential to impact cancer death and death rate projections. Second, the change in the number of cases between time periods has been allocated into changes because of risk, age structure, and population size. This allocation is arbitrary because the 3 components mutually affect each other. For example, if the population size increases, the effect of higher death rates (the risk component) will be larger than if the population size does not change. In the analysis of past trends, the first year (1975) was used as the reference year, following the approach in Canada (11). In the analysis of future trends, the final year (2020) was preferred from a preventive prospective (14). The consequence of using the final year as a reference rate is that the change in the number of deaths because of the combined effect of risk, age structure, and population size is attributed to risk, not demographics. If a future reduction in the risk of death can be achieved, the number of deaths from the combined effect of risk and demographics will be reduced.
